# Robotic purse-string suture technique for intracorporeal anastomosis using double-stapling technique in robotic resection of rectal and sigmoid colon cancer: a propensity score-matched analysis

**DOI:** 10.1186/s12893-024-02551-8

**Published:** 2024-09-05

**Authors:** Masayuki Hiraki, Kiminori Yanagisawa, Ryo Ikeshima, Taishi Hata, Kazumasa Komura, Asami Arita, Shinsuke Katsuyama, Go Shinke, Mitsuru Kinoshita, Yoshiaki Ohmura, Keijiro Sugimura, Toru Masuzawa, Yutaka Takeda, Kohei Murata

**Affiliations:** 1 Department of Surgery, Kansai Rosai Hospital, 3-1-69 Inabaso, Amagasaki, Hyogo 660-8511 Japan; 2 Division of Translational Research, Osaka Medical and Pharmaceutical University, 2-7 Daigaku-Machi, Takatsuki City, Osaka, 569-8686 Japan

**Keywords:** Robotic purse-string suture, Left-sided ICA, Rectal cancer, Robotic surgery

## Abstract

**Background:**

Robotic three-dimensional magnified visual effects and field of view stabilization have enabled precise surgical operations. Intracorporeal anastomosis in right-sided colorectal cancer surgery is expected to shorten operation times, avoid paralytic ileus, and shorten wound lengths; however, there are few reports of intracorporeal anvil fixation for intestinal anastomosis in left-sided colorectal cancer surgery. Herein, we introduce a simple, novel procedure for using robotic purse-string suture (RPSS) in intracorporeal anastomosis with the double-stapling technique in rectal and sigmoid cancer surgery and report short-term outcomes.

**Methods:**

From September 2022 to April 2024, 105 consecutive patients underwent robotic surgery with double-stapling technique anastomosis for rectal or sigmoid colon cancer at our institution. Their data were retrospectively analyzed. Intracorporeal anastomosis with the double-stapling technique using RPSS was performed in 26 patients (the RPSS group), while the double-stapling technique anastomosis with extracorporeal anvil fixation was performed in 79 patients (the EC group). A 1:1 propensity score-matched analysis was performed (matching criteria: sex, age, body mass index (BMI), tumor location and tumor size) using a caliper 0.3.

In the RPSS group, after tumor-specific or total mesorectal excision, specimens were extracted from the umbilical wound with simultaneous anvil placement in the body cavity. The oral colonic stump was robotically excised and robotically circumferentially stitched with 3–0 Prolene in all layers. After anvil insertion into the stump, the bowel wall of the colon was completely sewn onto the central rod of the anvil. Reconstructions were anastomosed using the double-stapling technique.

**Results:**

The matched cohort contained 23 patients in each group. The RPSS group had significantly less bleeding than the EC group (*p* = 0.038). Super-low anterior resection (SLAR) in the RPSS group had shorter total operative times than those in the EC group (*p* = 0.045). The RPSS group experienced no perioperative complications greater than Clavien–Dindo grade III or any anastomosis-related complications.

**Conclusions:**

The RPSS technique can be performed safely without any anastomosis-related complications and reduces the total operative times in SLAR and blood loss through total robotic surgery. This may be a useful modality for robotic colorectal surgery.

## Background

The robotic three-dimensional magnified visual effects and stabilized field of view have enabled precise surgical operations. Robot-assisted surgery has been increasingly performed for rectal cancer to address increasing social needs. Moreover, with the recent extension in the criteria indicating colon cancer in Japan, intracorporeal anastomosis (ICA) techniques for right-sided colectomy have gradually spread.

ICA has the merits of cosmesis and minimal invasiveness, achieving minimization of the range of colorectal mobilization, reduction of wound length, reduction of bleeding risk due to tissue traction, early recovery from postoperative intestinal peristalsis, and reduction of postoperative pain [1, 2]. This is particularly important in cases with high adhesion, as it contributes to shortening the operation time by minimizing the movement range of the intestinal tract and eliminating unnecessary adhesion detachments.

To date, most existing studies have reported an improvement in outcomes of laparoscopic right-sided ICA techniques compared with extracorporeal anastomosis (ECA) [3, 4]. Kelley et al. previously reported that robotic right colectomy with ICA is technically feasible, efficacious, oncologically acceptable, and safe to perform, with excellent short-term outcomes [5]. Moreover, Sorgato et al. compared laparoscopic and robotic approaches for right-sided colectomy with the ICA and concluded that intracorporeal ileocolic anastomosis is safe, faster, and easier to perform with robotic procedures [6].

During robotic left-sided surgery for colorectal cancer, most anastomoses are partially performed extracorporeally. The robot is undocked once to expose the resected specimen to the outside of the body, and an anvil is inserted into the proximal intestinal stump. The anastomosis is then completed laparoscopically or by redocking the robot, both of which can prolong the total surgical time.

Although intracorporeal anvil fixation for intestinal anastomosis in left-sided colorectal cancer surgery may also have some advantages, there are few reports have investigated that. In a study examining left-sided ICA for any disease process, Hollandsworth et al. suggested that the robotic stapled intracorporeal technique could be a technically feasible and safe option for intestinal anastomosis following left-sided colectomy [7]. In their anvil-forward technique, the anvil is first inserted into the oral intestine which is closed using a robotic stapler, and the tip of the anvil is then brought out near the staple line. Therefore, four double staples are used at the anastomotic site, resulting in an increased number of staple intersections and risk of anastomotic leakage.

Herein, we report a simple and novel technique of robotic purse-string suture (RPSS) without a stapler to perform ICA using the double-stapling technique (DST) in robotic surgery for rectal and sigmoid cancer. Purse-string suture technique has certainly been reported in many cases of laparoscopic surgery for the gastrointestinal tract, such as esophageal reconstruction, duodenal stump embedding, pancreaticoduodenectomy and appendicitis [8–11], and is undoubtedly very advantageous. However, to our knowledge, there have been no reports of purse-string suture related to DST anastomosis in robotic colorectal surgery.

## Methods

### Patients and study design

We performed this study according to the STROBE guidelines. In this retrospective study, we enrolled 105 consecutive patients diagnosed with rectal or sigmoid colon cancer who underwent robotic surgery with DST anastomosis at our institution between September 2022 and April 2024. Participants' ages ranged from 37 to 93 years. ICA with DST using RPSS was performed in 26 patients (the RPSS group), while DST anastomosis with extracorporeal anvil fixation was performed in 79 patients (the EC group). A 1:1 propensity score-matched analysis was performed (matching criteria: sex, age, body mass index (BMI), tumor location and tumor size). We compared perioperative outcomes among 23 patients in each group. The exclusion criteria were as follows: perforated cancer or cancer exceeding 80 mm on preoperative assessments; dementia; Performance Status (PS) of 3 or 4; severe obesity, defined as a body mass index (BMI) of > 35 kg/m^2^; previous abdominal polysurgery; and indications for emergency surgery. Preoperatively, all the patients received a combination of mechanical and chemical bowel preparations (kanamycin and metronidazole, respectively).

### Surgical settings and port placement

**Fig. 1:**
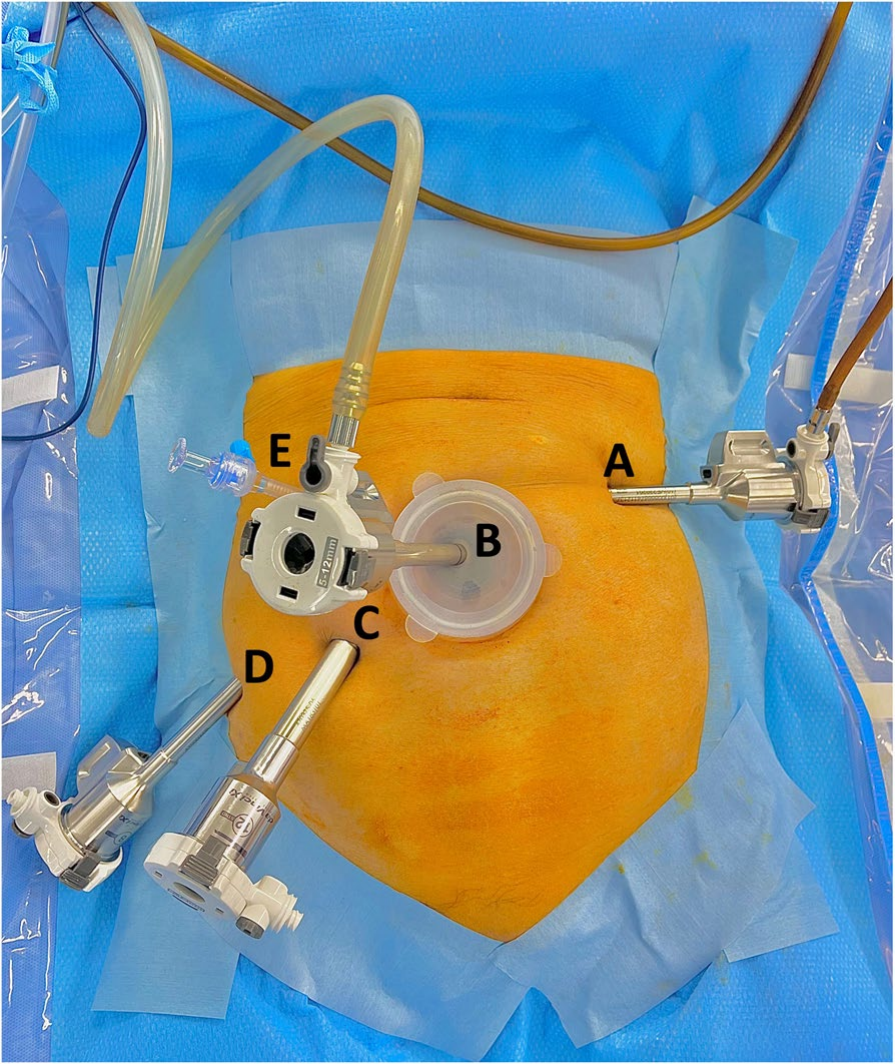
Trocar placement and instruments used for surgery. **A** 8**-**mm port, fenestrated bipolar forceps. **B** 8**-**mm port, robotic camera. **C** 12**-**mm port, monopolar curved scissors, vessel sealer, clip applier, SutureCut needle driver, and robotic stapler. **D** 8**-**mm port, tip-up fenestrated grasper. **E**: 5**-**mm laparoscopic assist port

In robotic surgery for rectal and sigmoid cancers, a small incision of 25 mm was first made in the umbilical site, and the access device and an 8-mm robotic port, which were used for the camera, were then inserted into the umbilical region. The umbilical incision was extended after removal of the specimen. After the abdomen was insufflated, an 8-mm (tip-up fenestrated grasper) and one 12-mm (monopolar curved scissors) robotic port were placed in the right lower quadrant. One 8-mm robotic port was placed in the left upper quadrant (fenestrated bipolar forceps) and a 5-mm port was placed in the right upper quadrant as an assist port. The da Vinci Xi robot system was docked on the patient’s left side with the instruments, with the boom facing the patient’s pelvis (Fig. 1). Monopolar curved scissors at the 12-mm port incision site were switched for the clip applier, vessel sealer, SutureCut needle driver, and robotic stapler, as necessary.

### RPSS surgical technique

**Fig. 2:**
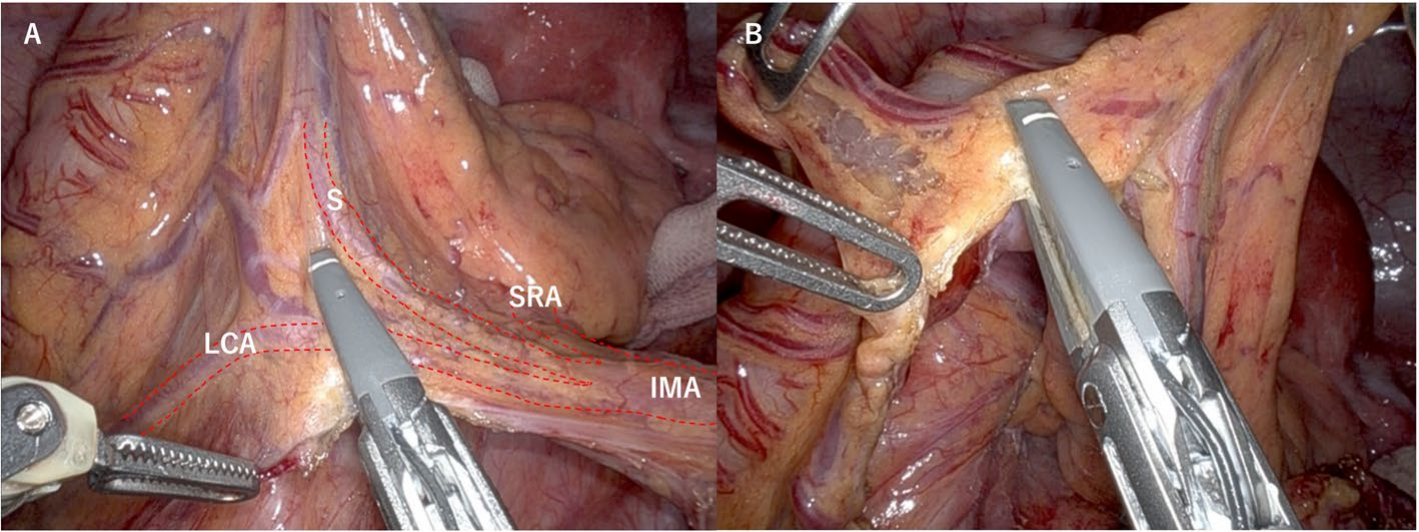
Dissection range of the oral mesentery. **A** For mesenteric resection on the oral side, the lymph nodes along IMA to SRA were dissected, and LCA was cut. **B** The mesentery was excised using a vessel sealer to an area of at least 10 cm proximal to the tumor. IMA, inferior mesenteric artery; SRA, superior rectal artery; LCA, left colic artery, S, sigmoid artery

Robotic resection of rectal or sigmoid cancer was performed with tumor-specific mesorectal excision or total mesorectal excision, followed by dissection of the oral and anal intestinal membranes. For mesenteric resection on the oral side, the lymph nodes along inferior mesenteric artery (IMA) to superior rectal artery (SRA) were dissected, and left colic artery (LCA) was cut (Fig. 2A). The mesentery was excised using a vessel sealer to an area of at least 10 cm proximal to the tumor (Fig. 2B).

**Fig. 3:**
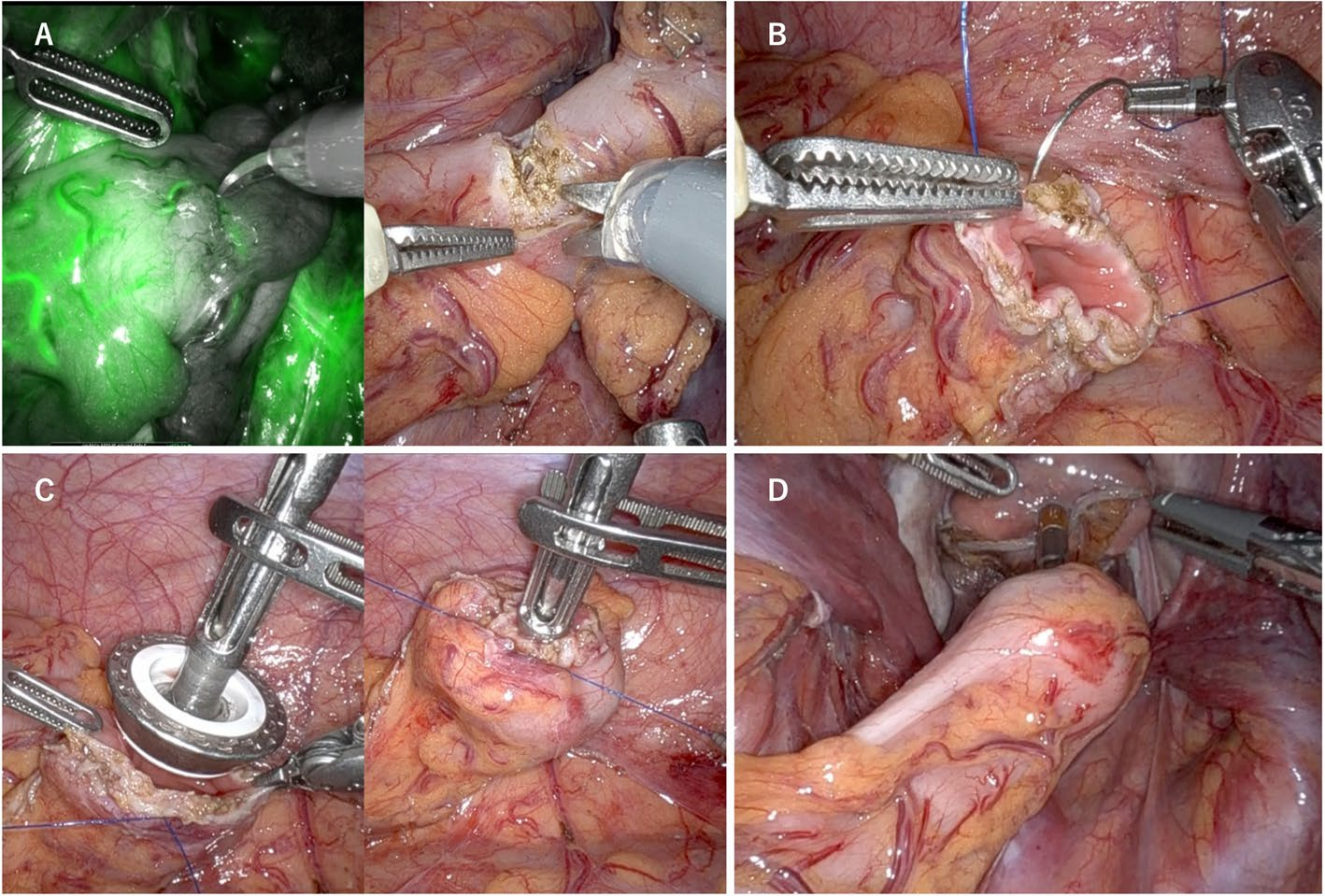
Surgical RPSS technique. **A** After blood flow confirmation using ICG, scissors excise the oral intestinal tract. **B** The oral colonic stump is robotically stitched with 3–0 Prolene circumferentially and to hold margins in all layers. **C** After anvil insertion into the hand-stitched colonic stump, the intestinal wall of the colon is completely sewn on the central rod of the anvil. **D** The reconstruction is finally anastomosed using DST. RPSS, robotic purse-string suture; ICG, indocyanine green; DST, double-stapling technique

The proximal colon and distal rectum were closed using robotic staplers following the demarcation line. The anvil was placed into the body cavity at the same time that the specimen was extracted from the umbilical wound. After confirming intestinal blood flow using the indocyanine green system, the oral intestinal tract was excised using scissors (Fig. 3A). The oral colonic stump was robotically hand-stitched with No. 3–0 Prolene circumferentially to secure the margins of all layers (Fig. 3B). After the anvil was inserted into the hand-stitched colonic stump using a tip-up fenestrated grasper, the intestinal wall of the colon was completely sewn onto the central rod of the anvil (Fig. 3C). The reconstruction was finally anastomosed using the DST with the ECHELON CIRCULAR® Powered Stapler (Ethicon, Somerville, NJ, USA) (Fig. 3D).

### Statistical analyses

All data were collected and analyzed using JMP Pro (version 17.1; SAS Institute Inc., Cary, NC, USA). Numeric data are presented as median (interquartile range [IQR]) or mean (standard deviation [SD]). Continuous variables were compared using the Mann–Whitney U test, whereas categorical variables were compared using the Pearson χ^2^ test. Differences with *p*-values ≤ 0.05 were considered statistically significant.

### Propensity score matching

We performed propensity score matching analysis in order to minimize possible confounders between the RPSS and EC groups. We used JMP Pro (version 17.1; SAS Institute Inc., Cary, NC, USA) to generate linear propensity score values using the logistic regression method. The RPSS and EC groups were then paired 1:1 on these propensity scores. A standard caliper size of 0.3 × log [SD of the propensity score] was used. Standardized differences were assessed before and after matching to evaluate the balance of covariates.

## Results

In the matched cohort after a 1:1 propensity score matching, the RPSS and EC groups had similar patient and tumor characteristics regarding sex (*p* = 1.0), age (60 vs. 64 years, respectively; *p* = 0.82), BMI (23 vs. 23 kg/m^2^, respectively; *p* = 0.62), tumor location (*p* = 0.91), and tumor size (30 vs. 30 mm, respectively; *p* = 0.91) (Table 1).

**Table 1:** Patients and tumor characteristics

Unmatched cohort (*n* = 105)			
Variables	RPSS group ( *n* = 26 )	EC group ( *n* = 79 )	*p *value
Sex			
Male/Female	16/10	48/31	1.0
Age^a^ years	60 (17)	67 (20)	0.37
BMI^a^ kg/m^2^	20 (6.1)	20 (7.1)	0.29
Tumor location			
S/RS/Ra/Rb	5/7/5/9	6/23/26/24	0.27
Tumor size^a^ mm	30 (33)	40 (29)	0.60
Matched cohort after 1:1 Propensity score matching (*n* = 46)			
Variables	RPSS group ( *n* = 23 )	EC group ( *n* = 23 )	*p *value
Sex			
Male/Female	13/10	13/10	1.0
Age^a^ years	60 (17)	64 (18)	0.82
BMI^a^ kg/m^2^	23 (4.9)	23 (3.7)	0.62
Tumor location			
S/RS/Ra/Rb	5/6/4/8	4/8/3/8	0.91
Tumor size^a^ mm	30 (30)	30 (30)	0.91

*BMI* Body mass index

^a^Values are median (IQR)

The surgical outcomes and oncological clearance were compared between the RPSS and EC groups (Table 2). In the matched cohort, surgical operations (*p* = 0.93), ileostomy frequency (30% vs. 39%, respectively; *p* = 0.54), total operation times (293 vs. 337 min, respectively; *p* = 0.36), and leak test positive rates (0% vs. 0%, respectively) were not significantly different between the two groups. Despite the equality of total operative times, super-low anterior resection (SLAR) in the RPSS group had shorter total operative times compared with that in the EC group (401 vs. 649 min, respectively; *p* = 0.045). The RPSS group had significantly less estimated blood loss (5.3 vs. 23 mL, respectively; *p* = 0.038) than the EC group. Umbilical incision length (30 vs. 32 mm, respectively; *p* = 0.15), the number of lymph nodes harvested (16 vs. 19, respectively; *p* = 0.21), frequency of lateral lymph node dissection (9.0% vs. 26%, respectively; *p* = 0.11), circumferential resection margin-positive rates (0% vs. 0%, respectively), and p-staging status (*p* = 0.63) were similar between the two groups.

**Table 2:** Surgical outcomes and Oncologic clearance of all patients

Unmatched cohort (*n = *105)			
Variables	RPSS group ( *n* = 26 )	EC group ( *n* = 79 )	*p *value
*Surgical outcomes*			
Surgical technique			
Sigmoidectomy/AR/LAR/SLAR	4/8/7/7	6/17/30/26	0.54
Ileostomy N (%)	9 (35)	29 (37)	1.0
Total operation times			
All surgical techniques^a^ min	294 (131)	340 (193)	0.19
Sigmoidectomy, AR^a^ min	256 (90)	242 (100)	1.0
LAR^a^ min	326 (213)	373 (113)	0.62
SLAR^a^ min	361 (154)	517 (280)	0.064
Blood loss^b^ mL	5 (9.5)	19 (40)	0.014
Length of umbilical incision^b^ mm	29 (0.57)	31 (0.63)	0.047
Leak test positive N (%)	0 (0)	0 (0)	
*Oncologic clearance*			
Number of lymph nodes harvested^a^	15 (8.5)	17 (13)	0.34
Lateral lymph node dissection N (%)	3 (12)	11 (14)	1.0
CRM positive N (%)	0 (0)	1 (1.3)	1.0
pStaging			
0/I/II/III/IV	0/10/7/7/2	1/27/24/23/4	0.94
Matched cohort after a 1:1 Propensity score matching (*n = *46)			
Variables	RPSS group ( *n* = 23 )	EC group ( *n* = 23 )	*p *value
*Surgical outcomes*			
Surgical technique			
Sigmoidectomy/AR/LAR/SLAR	4/7/6/6	4/6/5/8	0.93
Ileostomy N (%)	7 (30)	9 (39)	0.54
Total operation times			
All surgical techniques^a^ min	293 (116)	337 (279)	0.36
Sigmoidectomy, AR^a^ min	268 (96)	252 (106)	0.86
LAR^a^ min	279 (143)	334 (180)	0.58
SLAR^a^ min	401 (210)	649 (309)	0.045
Blood loss^b^ mL	5.3 (10)	23 (36)	0.038
Length of umbilical incision^b^ mm	30 (0.56)	32 (0.53)	0.15
Leak test positive N (%)	0 (0)	0 (0)	
*Oncologic clearance*			
Number of lymph nodes harvested^a^	16 (9)	19 (18)	0.21
Lateral lymph node dissection N (%)	2 (8.7)	6 (26)	0.11
CRM positive N (%)	0 (0)	0 (0)	
pStaging			
0/I/II/III/IV	0/9/6/7/1	0/8/9/6/0	0.63

*AR* Anterior resection, *LAR* Low anterior resection, *SLAR* Super low anterior resection, *CRM* Circumferential resection margin

^a^Values are median (IQR)

^b^Values are mean (SD)

The RPSS and EC groups in the matched cohort had similar postoperative outcomes regarding the time to start eating (4 vs. 3 days, respectively; *p* = 061), time before first flatus (2 vs. 2 days, respectively; *p* = 0.84), postoperative hospital stay (9 vs. 10 days, respectively; *p* = 0.91), visual analogue scale score on postoperative day 0 (3.5 vs. 4 scores, respectively; *p* = 0.50), frequency of readmission (0% vs. 0%, respectively), and all complications (Clavien–Dindo grade ≥ I). In the RPSS group, no patient experienced perioperative complications above Clavien-Dindo grade III or any anastomosis-related complications (Table 3).

**Table 3:** Postoperative outcomes and complications

Unmatched cohort (*n = *105)			
Variables	RPSS group ( *n =* 26 )	EC group ( *n =* 79 )	*p *value
*Postoperative outcomes*			
Time to start eating^a^ day	4 (3)	3 (3)	0.82
Time before first flatus^a^ day	2 (2)	2 (2)	0.37
Postoperative hospital stay^a^ day	9 (6.5)	10 (8)	0.61
VAS score on POD0^a^	4 (3)	4 (4)	0.56
Readmission, N (%)	0 (0)	0 (0)	
Complications (Clavian Dindo grade ≧ I)			
Wound infection, N (%)	1 (3.8)	2 (2.5)	1.0
Urinary tract infection, N (%)	0 (0)	3 (3.8)	0.57
Lymphorrhoea, N (%)	2 (7.7)	2 (2.5)	0.26
Ileus, N (%)	1 (3.8)	3 (3.8)	1.0
Anastomotic leakage, N (%)	0 (0)	1 (1.3)	1.0
Abdominal incisional hernia, N (%)	1 (3.8)	2 (2.5)	0.74
Matched cohort after a 1:1 Propensity score matching (*n = *46)			
Variables	RPSS group ( *n =* 23 )	EC group ( *n =* 23 )	*p *value
*Postoperative outcomes*			
Time to start eating^a^ day	4 (3)	3 (2)	0.61
Time before first flatus^a^ day	2 (2)	2 (1)	0.84
Postoperative hospital stay^a^ day	9 (8)	10 (11)	0.91
VAS score on POD0^a^	3.5 (3)	4 (2)	0.50
Readmission, N (%)	0 (0)	0 (0)	
Complications (Clavian Dindo grade ≧ I)			
Wound infection, N (%)	1 (4.4)	1 (4.4)	1.0
Urinary tract infection, N (%)	0 (0)	1 (4.4)	0.31
Lymphorrhoea, N (%)	2 (8.7)	1 (4.4)	0.55
Ileus, N (%)	1 (4.4)	2 (8.7)	0.55
Anastomotic leakage, N (%)	0 (0)	0 (0)	
Abdominal incisional hernia, N (%)	1 (4.4)	0 (0)	0.31

*VAS* Visual analogue scale, *POD* Postoperative day

^a^Values are median (IQR)

## Discussion

Laparoscopic surgery for colorectal cancer has been developed to achieve minimal invasiveness and improved cosmesis [12]. Reduced-port surgery, which seeks to reduce the size of wounds; single-incision laparoscopic surgery, which removes the ports on the flank and centralizes wounds of the abdominal wall onto the umbilical wound [13]; and natural orifice transluminal endoscopic surgery, which minimizes wounds on the body surface using physiological orifices, are prominent examples.

The ICA technique also offers the advantages of minimal invasiveness and improved cosmesis, including the minimization of the range of colorectal mobilization, reduction in wound length, and early recovery from postoperative intestinal peristalsis [1, 2]. Although ICA has many advantages, most DST anastomoses are partially performed extracorporeally in laparoscopic left-sided surgery for colorectal cancer. This is primarily because management of the anvil of a circular stapling device can be technically challenging in total laparoscopic surgery due to the intricate procedures required to fix it to the oral colonic stump. In this context, Liang et al. introduced a new laparoscopic manual binding technique as a relatively simple method of tying the anvil to the oral stump of the intestine, even during laparoscopic surgery. They reported that the ICA technique in total laparoscopic surgery for high-mid rectal cancer was safe and feasible [14].

Robot-assisted surgery is being rapidly adopted, and can overcome the intrinsic limitations of laparoscopic surgery [15], owing to the robotic three-dimensional magnifying visual effect, stabilized field of vision, superior range of motion, and motion scaling. However, in even robot-assisted surgery, most DST anastomoses remain partially extracorporeal, which can increase both the effort of the assistant and the total surgical time. As precise robotic operability makes securing the anvil to the oral colonic stump easier, our RPSS technique for conducting left-sided ICA using DST resolves these problems. According to our preliminary results, SLAR using RPSS had shorter total operative times compared with extracorporeal anvil fixation (*p* = 0.045) (Table 2). This result suggests that the deeper the pelvic floor operation, the more smoothly the anastomosis can be performed through total robotic surgery. If the anvil is fixed externally, the anastomosis is then performed laparoscopically. As with SLAR, the longer the surgery takes, the more noticeable are air leaks from the side of the trocar, hand shake from handling the robot camera, and difficulty in performing the anastomosis.

No patients required splenic flexure mobilization or experienced perioperative complications greater than Clavien–Dindo grade III or any anastomosis-related complications (Table 3).

Our results confirmed that RPSS-ICA is a safe and feasible procedure. However, this study had some limitations. First, this study included only preliminary data from a small number of patients. Second, all patients were recruited from a single institution. A prospective randomized controlled trial with a larger number of patients should be conducted in the future to further validate our results. Third, robotic hand stitching can result in an uneven suturing depth and spacing. Ideally, purse string instrument forceps should be developed that can easily be handled inside the body cavity. Fourth, surgeons in both groups were not completely identical. RPSS was performed by three robotic surgeons, including two proctors, whereas EC was performed by four surgeons, including three proctors. All surgeons who performed RPSS are included in those who performed EC.

## Conclusions

RPSS for ICA in total robotic resection of rectal and sigmoid cancers may be a promising modality with many advantages that simplifies the operation, reduces the burden on assistants, and minimizes operative time and blood loss. However, large-scale prospective studies are needed to validate our findings.

## Data Availability

No datasets were generated or analysed during the current study.

## Abbreviations

BMI: Body mass index
DST: Double-stapling technique
ECA: Extracorporeal anastomosis
ICA: Intracorporeal anastomosis
IQR: Interquartile range
SD: Standard deviation
RPSS: Robotic purse-string suture
SLAR: Super-low anterior resection

## Availability of data and materials

Data will be made available from the corresponding author, MH, on reasonable request.
